# Rural–urban differences in mortality from concurrent heart failure and cancer, 2011–2020

**DOI:** 10.1038/s41598-026-62290-z

**Published:** 2026-07-22

**Authors:** Anum Zehra, Joseph J. Shearer, Maryam Hashemian, Gretell Henríquez-Santos, Jungnam Joo, Jacqueline B. Vo, Véronique L. Roger

**Affiliations:** 1 Heart Disease Phenomics Laboratory, Epidemiology and Community Health Branch, National Heart, Lung and Blood Institute, National Institutes of Health, 10 Center Drive, Building 10, Room 8N228, Bethesda, MD 20814 USA; 2 School of Medicine, University of Maryland, Baltimore, MD USA; 3 Office of Biostatistics Research, National Heart, Lung, and Blood Institute, National Institutes of Health, Bethesda, MD USA; 4 Radiation Epidemiology Branch, Division of Cancer Epidemiology & Genetics, National Cancer Institute, Bethesda, MD USA

**Keywords:** Heart failure, Cancer, Comorbidity, Rurality, Mortality, Cancer, Cardiology, Diseases, Health care, Medical research, Oncology, Risk factors

## Abstract

Rural populations experience higher mortality rates than urban populations for both heart failure (HF) and cancer, however, mortality involving both conditions has not been well characterized. We evaluated national trends and demographic differences in HF-related and HF + cancer–related deaths and assessed rural–urban disparities. Using U.S. death certificate data from 2011 to 2020, we calculated age-adjusted mortality rates (AAMRs) per 100,000 persons for HF-related and HF + cancer–related deaths overall and stratified by rurality. During the study period, 3,453,655 HF-related deaths occurred, of which 274,715 (8%) also involved cancer. AAMRs increased over time for both outcomes. Rural areas consistently demonstrated higher mortality rates than urban areas for HF-related deaths (143.2 vs 110.1 per 100,000) and HF + cancer–related deaths (11.6 vs 8.8 per 100,000). Rural–urban disparities were evident across sex, age, and race/ethnicity groups. The largest rural-to-urban AAMR ratios were observed for lung/bronchus cancer (1.41), colorectal cancer (1.39), and leukemia (1.32). Mortality associated with both HF and cancer disproportionately affects rural populations. These findings highlight a growing dual burden of cardiovascular disease and cancer in rural communities and underscore the need for targeted prevention and care strategies addressing multimorbidity in these settings.

## Introduction

Heart disease and cancer together account for nearly 40% of all deaths in the United States (U.S.)^1^. Heart failure (HF) alone affects 6.7 million adults and contributes to over 450,000 deaths annually^2^. In addition to its substantial burden, HF is associated with an increased risk of developing cancer, which has emerged as the leading non-cardiovascular cause of death among individuals with HF^3,4^. Mortality rates for both cancer and HF are rising, with marked differences across sex and racial and ethnic groups^5^. Concurrently, a growing number of individuals are living with both HF and cancer, in part due to improved survival and the long-term cardiotoxic effects of cancer therapies^2^. Together, these trends underscore the importance of understanding patterns of mortality related to both conditions, including when they co-occur.

HF and cancer share a range of risk factors, including comorbid conditions (e.g., hypertension, obesity, diabetes), health behaviors (e.g., diet, smoking), environmental exposures, and broader social determinants of health (e.g., race, sex)^6–9^. Many of these factors disproportionately affect rural populations, who comprise approximately 20% of the U.S. population and experience a higher burden of disease and worse outcomes^10^. Rural–urban disparities in mortality continue to widen, particularly among adults aged 25 to 54 years^11^. Although rural residence has been associated with higher mortality from HF and cancer individually^12–16^, less is known about whether rural populations are at increased risk of mortality related to the co-occurrence of these conditions.

To address this gap, we examined U.S. death certificate data from 2011 through 2020 to: (1) characterize national trends and demographic patterns in deaths involving both HF and cancer, in the context of overall HF-related mortality; and (2) assess differences in these patterns by rurality.

## Methods

### Data source and study population

We analyzed U.S. mortality data from 2011 to 2020 using the Centers for Disease Control and Prevention Wide-ranging Online Data for Epidemiologic Research (CDC WONDER)^17^. Final Multiple Cause of Death public-use file, which contains death certificate data for U.S. residents from all counties, including demographic characteristics (age, sex, race/ethnicity), one underlying cause of death, and up to 20 contributing causes, classified according to the International Classification of Diseases, Tenth Revision (ICD-10)^18^. Mortality data were available through 2020 at the time of analysis.

We included decedents aged ≥ 15 years. Age was categorized as 15–44, 45–64, 65–84, and ≥ 85 years. Race/ethnicity was categorized as Hispanic or Latino; non-Hispanic White (NHW); non-Hispanic Black (NHB); non-Hispanic American Indian or Alaska Native (NHAIAN); and non-Hispanic Asian or Pacific Islander (NHAPI). Individuals with ethnicity recorded as “not stated” were excluded from race/ethnicity-stratified analyses. The study was exempt from Institutional Review Board approval because it used publicly available, de-identified data.

### Exposure definition (rural–urban classification)

The county rural–urban status was defined using the 2013 National Center for Health Statistics Urban–Rural Classification Scheme for Counties within CDC WONDER.^19^ Urban areas included large central, large fringe, medium, and small metropolitan counties; rural areas included micropolitan and noncore counties.

### Outcome definition (related-cause mortality)

A “related” cause of death was defined as any mention (underlying or contributing) on the death certificate. Because multiple causes may be recorded, categories were not mutually exclusive.

Heart failure (HF)–related deaths were identified using ICD-10 codes I11.0, I13.0, I13.2, and I50.x listed anywhere on the death certificate. HF + cancer–related deaths were defined as deaths with concurrent HF codes and any malignant neoplasm ICD-10 codes (C00–C97).

HF + cancer deaths were further stratified by cancer type using ICD-10 codes: lung/bronchus (C34), colorectal (C18–C20), pancreatic (C25), breast (C50), prostate (C61), liver and intrahepatic bile duct (C22), leukemia (C91–C95), and non-Hodgkin lymphoma (C82–C85). These cancers were selected based on their contribution to overall U.S. cancer mortality according to the 2024 Surveillance, Epidemiology, and End Results report^20^. Similar ICD-10 coding approaches have been used in previous analyses of HF-related mortality and cancer-related mortality using CDC WONDER data^21,22^.

### Covariates

Covariates included age group, sex, race/ethnicity, and rural–urban classification.

### Statistical analysis

Crude and age-adjusted mortality rates (AAMRs) per 100,000 persons were calculated and standardized to the 2000 U.S. standard population^23^. Crude rates were reported for individuals aged ≥ 85 years because CDC WONDER provides age-adjusted rates only when multiple age groups are selected.

Rural-to-urban mortality rate ratios were calculated overall and within subgroups. Temporal trends were assessed using the Joinpoint Regression Program (Version 4.9.1.0; National Cancer Institute). Models allowed up to two joinpoints. Annual percent change (APC) was estimated using Monte Carlo permutation methods to identify statistically significant changes over time, which accounts for estimated variation across each year and time point to identify significant changes in trends^24^. 

## Results

Between 2011 and 2020, 3,453,655 HF-related deaths occurred among U.S. decedents aged ≥ 15 years. Of these, 274,715 (8%) also involved cancer (Table 1). The proportion of HF-related deaths with concurrent cancer remained stable over time (7.6%–8.4% annually).

**Table 1:** Decedent characteristics by cause of death.

Categories	HF-related			HF + cancer-related		
	N	%	AAMR (95% CI)	N	%	AAMR (95% CI)
Overall	3,453,655	100	115.68 (115.56–115.81)	274,715	100	9.26 (9.23–9.30)
Age (years)						
15–44	32,985	0.96	2.80 (2.77–2.83)	1,414	0.51	0.11 (0.10–0.11)
45–64	317,801	9.20	34.23 (34.11–34.35)	26,657	9.70	2.79 (2.76–2.82)
65–84	1,419,128	41.09	364.58 (363.98–365.19)	144,352	52.55	36.79 (36.59–36.98)
*85 +	1,683,741	48.75	2682.33 (2,678.28–2,686.38)	102,292	37.24	162.96 (161.96–163.96)
Sex						
Male	1,654,498	47.91	137.18 (136.96–137.39)	155,738	56.69	12.82 (12.76–12.89)
Female	1,799,157	52.09	99.78 (99.63–99.93)	118,977	43.31	6.86 (6.82–6.90)
Race/Ethnicity						
NH AIAN	18,071	0.52	113.03 (111.32–114.74)	1,461	0.53	9.14 (8.66–9.63)
NH API	65,993	1.91	52.33 (51.93–52.74)	4,965	1.81	3.88 (3.77–3.99)
NH Black	352,175	10.20	130.15 (129.71–130.59)	28,104	10.23	10.36 (10.23–10.48)
NH White	2,830,823	81.97	119.87 (119.73–120.01)	227,139	82.68	9.76 (9.72–9.80)
Hispanic/Latino	179,550	5.20	76.22 (75.86–76.58)	12,551	4.57	5.30 (5.21–5.40)
Urbanization						
Urban	2,729,840	79.04	110.08 (109.95–110.21)	215,991	78.62	8.78 (8.75–8.82)
Rural	723,815	20.96	143.16 (142.83–143.50)	58,724	21.38	11.57 (11.47–11.66)
Years						
2011–2015	1,537,448	44.52	108.85 (108.68–109.03)	119,975	43.68	8.61 (8.56–8.66)
2016–2020	1,916,207	55.48	121.82 (121.65–121.99)	154,740	56.32	9.86 (9.81–9.91)

AAMR, Age-Adjusted Mortality Rate; AIAN, American Indian or Alaskan Native; API, Asian or Pacific Islander; NH, non-Hispanic. ^*^Crude rates only available for 85 + year-old group.

**Fig. 1:**
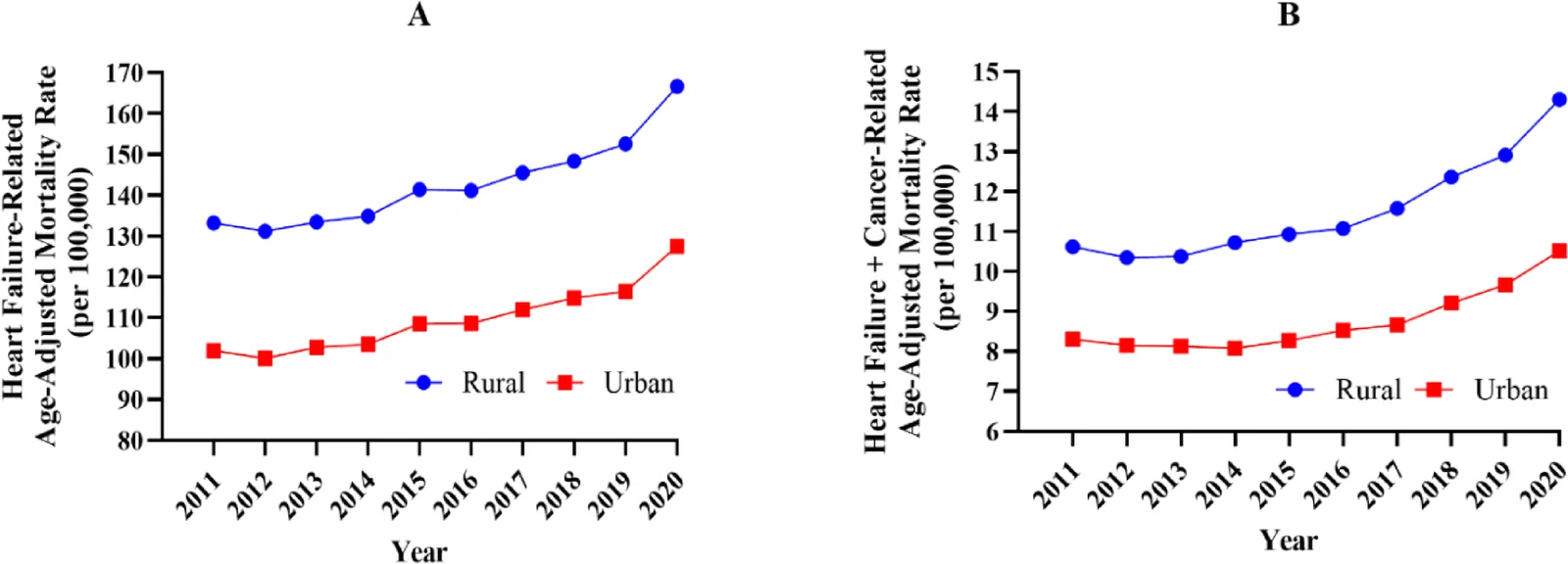
Rural and urban mortality from 2011 to 2020 for heart failure-related (**A**) and Heart Failure + Cancer-Related (**B**) Deaths.

Both HF-related and HF + cancer–related deaths increased during the study period (Fig. 1). The overall age-adjusted mortality rate (AAMR) was substantially higher for HF-related deaths (115.7; 95% CI: 115.6–115.8) than for HF + cancer–related deaths (9.3; 95% CI: 9.2–9.3). Compared with HF-related deaths overall, HF + cancer–related deaths included a lower proportion of women (43.3% vs 52.1%) and fewer decedents aged ≥ 85 years (37.2% vs 48.8%). However, men had higher AAMRs than women for both outcomes. The racial and ethnic distribution was similar between groups.

Although rural residents accounted for fewer than 25% of deaths, rural AAMRs were consistently higher than urban AAMRs for both HF-related (143.2; 95% CI: 142.8–143.5 vs 110.1; 95% CI: 110.0–110.2) and HF + cancer–related deaths (11.6; 95% CI: 11.5–11.7 vs 8.8; 95% CI: 8.8–8.8).

HF-related mortality increased in rural counties from 2011 to 2018 (APC: 1.7; 95% CI: 0.9–2.5), with a sharper rise from 2018 to 2020 (APC: 6.1; 95% CI: 0.8–11.6). Urban counties experienced a steady increase from 2011 to 2020 (APC: 2.4; 95% CI: 1.8–3.1). HF + cancer–related mortality increased beginning in 2016 in rural counties (APC2016–2020: 6.5; 95% CI: 4.4–8.8) and in 2017 in urban counties (APC2017–2020: 7.0; 95% CI: 3.9–10.3), following earlier periods of relative stability.

**Fig. 2:**
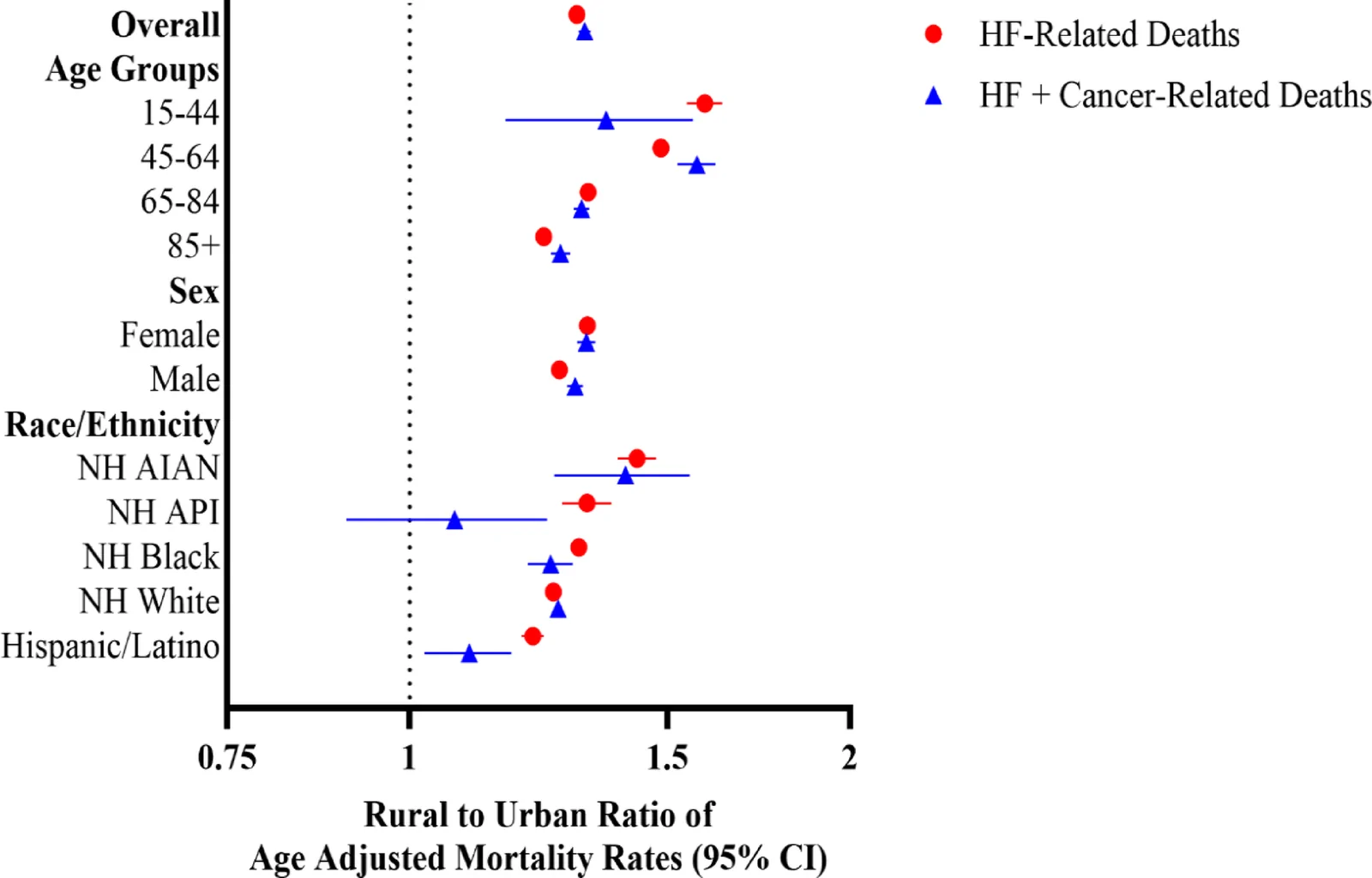
Rural-to-urban age-adjusted mortality rate ratios. NH, non-Hispanic; AIAN, American Indian or Alaskan Native; API, Asian or Pacific Islander.

Rural-to-urban AAMR ratios exceeded 1.0 overall and across all age, sex, and race/ethnicity subgroups for both outcomes (Fig. 2). Among HF-related deaths, the largest disparity was observed in individuals aged 15–44 years (1.59; 95% CI: 1.54–1.64), whereas for HF + cancer–related deaths, the largest disparity occurred among those aged 45–64 years (1.57; 95% CI: 1.52–1.62). Non-Hispanic American Indian or Alaska Native individuals had the highest rural-to-urban AAMR ratios for both HF-related (1.43; 95% CI: 1.39–1.47) and HF + cancer–related deaths (1.40; 95% CI: 1.26–1.55).

For most subgroups, rural–urban disparities were comparable between HF-related and HF + cancer–related deaths. Notable heterogeneity was observed among individuals aged 15–44 years and non-Hispanic Asian or Pacific Islander individuals, who demonstrated > 17% greater rural–urban disparities for HF-related compared with HF + cancer–related deaths.

When stratified by the eight leading cancer types, rural AAMRs exceeded urban AAMRs across all categories (Table 2). The largest rural-to-urban ratios were observed for lung/bronchus cancer (1.41; 95% CI: 1.38–1.43), colorectal cancer (1.39; 95% CI: 1.35–1.43), and leukemia (1.32; 95% CI: 1.27–1.36).

**Table 2:** Rural to urban AAMR ratios by cancer type among HF + cancer-related deaths.

Cancer Type	Rural		Urban		AAMR Ratio
	N	AAMR (95% CI)	N	AAMR (95% CI)	
Bronchus/Lung	12,735	2.49 (2.45–2.54)	43,243	1.77 (1.76–1.79)	1.41 (1.38–1.43)
Colorectal	5,700	1.12 (1.09–1.15)	19,739	0.80 (0.79–0.81)	1.39 (1.35–1.43)
Leukemia	4,234	0.84 (0.81–0.86)	15,521	0.64 (0.63–0.65)	1.32 (1.27–1.36)
Prostate	6,965	1.37 (1.34–1.41)	25,890	1.05 (1.04–1.06)	1.31 (1.27–1.34)
Breast	5,237	1.05 (1.02–1.08)	21,065	0.84 (0.83–0.85)	1.26 (1.22–1.30)
Pancreas	1,788	0.34 (0.32–0.35)	7,076	0.28 (0.27–0.28)	1.22 (1.16–1.29)
Non–Hodgkin lymphoma	3,416	0.67 (0.65–0.70)	13,687	0.57 (0.56–0.58)	1.19 (1.14–1.23)
Liver/Intrahepatic duct	1,365	0.27 (0.25–0.28)	5,850	0.23 (0.23–0.24)	1.15 (1.08–1.22)

## Discussion

Our findings provide new insight into the association between rural residence and mortality involving heart failure (HF), both independently and in combination with cancer. Since 2011, mortality rates for HF and HF + cancer have increased, with persistent and substantial disparities between rural and urban populations. These rural–urban differences were observed across demographic subgroups and cancer types. The widening gap underscores the growing burden of HF and cancer comorbidity in the United States and highlights the disproportionate impact on rural communities.

### HF-related and HF + cancer-related deaths

HF + cancer-related deaths comprised 8% of overall HF-related deaths and have steadily increased since 2011. This pattern is consistent with prior reports^5^, indicating that prior declines in HF + cancer-related mortality have largely plateaued or reversed in recent years. Heart disease and cancer share multiple risk factors, including cardiometabolic conditions such as obesity, hypertension, diabetes, and dyslipidemia. In addition, certain cancer therapies—notably specific chemotherapeutic agents and radiation—are associated with cardiotoxic effects that may increase the risk of subsequent heart disease among cancer survivors. Structural determinants, including differences in access to preventive care, specialty services, and timely treatment, may further shape the burden and outcomes of both conditions, particularly across geographic settings.

Prior work, however, has not evaluated these patterns in the context of overall heart failure–related mortality or examined differences by rurality. In the present analysis, we found that mortality rates were consistently higher in rural compared with urban populations for both heart failure–related deaths and deaths involving both heart failure and cancer. Similar disparities have been reported for cardiovascular mortality among cancer survivors, suggesting that these patterns may extend to populations in whom both conditions co-occur^25^. Several factors may contribute to this “rurality penalty,” although they could not be evaluated in the present study, including differences in the distribution of the cardiology workforce, in healthcare utilization, uptake of guideline-directed therapies, and availability of cardio-oncology services^26–28^. These factors represent plausible hypotheses that warrant further investigation.

### Rurality

Rural mortality rates were higher across all major demographic groups for both heart failure–related deaths and deaths involving both heart failure and cancer. Overall patterns were largely consistent between these outcomes, with some notable distinctions. Among younger decedents (15–44 years), the rural–urban gap was more pronounced for heart failure–related deaths than for deaths involving both conditions. The reasons for this difference remain unclear. One possible explanation is that temporal trends in cause-specific mortality diverge in younger populations; for example, prior studies have reported declining cancer mortality alongside increasing heart disease mortality among rural adults aged 25–54 years^11^. Another consideration is that patterns of care, including transfer to urban centers for more complex conditions, may differ by age group and could influence place-of-death classification^29^.

Among NHAPI populations, the rural–urban gap decreased when cancer was included as a contributory cause of death. Potential explanations include differences in behavioral risk factors and cancer screening patterns, as suggested in prior studies^30^. It is also important to note that aggregation of Asian and Pacific Islander populations may obscure important heterogeneity, as recent analyses have demonstrated substantial variation across these groups^31^. In contrast, NHAIAN populations experienced the largest rural–urban disparities for both HF-related and HF + cancer-related deaths, consistent with prior reports across cardiovascular and cancer outcomes^32^. Differences in socioeconomic factors and health behaviors have been described in these populations and may represent additional hypotheses to explain these patterns^10,33^.

### HF + cancer types by rurality

Rural mortality rates were consistently higher across all cancer types examined. Heart disease and cancer share a broad set of overlapping risk factors that likely contribute to their co-occurrence. Cardiometabolic conditions—including obesity, hypertension, diabetes, and dyslipidemia—are well-established drivers of both cardiovascular disease and multiple cancers. In addition, cancer therapies, such as certain chemotherapeutic agents and radiation, can have cardiotoxic effects, increasing the risk of subsequent heart disease among cancer survivors.

Structural factors, including disparities in access to preventive care, specialty services, and timely treatment, may further influence the burden and outcomes of both conditions, particularly across geographic settings. Moreover, shared risk factors such as smoking and lower uptake of screening—which vary by rurality—may help explain the observed patterns. Together, these overlapping biological and structural determinants provide a framework for understanding the intersection of heart disease and cancer and underscore key priorities for future investigation^5,34,35^. Differences in access to healthcare services and specialty care have also been documented between rural and urban populations and may play a role. For example, rural residents with hematologic malignancies are more likely to die at home than urban residents^36^, which may influence mortality patterns, including the relatively high rural–urban ratio for leukemia-related deaths in this study. Taken together, these findings demonstrate consistent rural–urban disparities in mortality involving both heart failure and cancer. The factors outlined above provide a set of testable hypotheses for future studies aimed at elucidating the underlying drivers of these inequities.

### Strengths and limitations

Our study has several strengths. Death certificate data were available on nearly all deaths in the U.S. using a standardized and comprehensive approach. While CDC WONDER provides limited covariate information, we were able to assess rurality using well-defined categories designed by the National Center for Health Statistics (2013), which increases confidence in our findings of higher mortality rates among rural counties. Furthermore, including HF and cancer as contributory causes of death (regardless of the underlying cause) allowed for a comprehensive approach to analyze the collective burden of these diseases. This approach may improve accuracy as the underlying cause of HF may often be misclassified^37^.

Several limitations warrant consideration. Deaths were ascertained using ICD-10 codes and may be subject to misclassification. Rural–urban classification was based on place of death, which may not reflect lifetime exposure. County urban–rural assignments obtained from the 2013 and 2006 schemes were similar. As with any observational analysis, the study is susceptible to residual confounding from unmeasured factors. The multiple cause of death database does not contain treatment information, cancer stage, heart failure severity, or other patient-level clinical characteristics, limiting our ability to evaluate mechanisms underlying observed trends. In addition, analyses were conducted at the population level and therefore should not be interpreted as reflecting individual-level associations between rurality and outcomes. Finally, we did not evaluate the potential impact of the COVID-19 pandemic, during which prior studies have documented increases in cardiovascular mortality, particularly in rural populations^38,39^.

## Conclusion

In this analysis of U.S. death certificate data, mortality involving both heart failure and cancer was consistently higher in rural compared with urban populations across demographic groups and cancer types. Parallel patterns were observed for heart failure alone as well as for coexisting heart failure and cancer, with persistent and pervasive rural–urban disparities.

These findings highlight substantial and enduring geographic inequities in mortality and reinforce the need for continued surveillance and focused investigation to better understand and address their underlying drivers. Potential strategies to reduce these disparities include expanding access to multidisciplinary cardio-oncology services, telehealth-enabled cardiovascular and cancer care, and community-based programs that improve prevention and management of multimorbidity in rural populations.

## Data Availability

All data are publicly available through the CDC WONDER website (https://wonder.cdc.gov/mcd.html).

## Abbreviations

AAMR: Age-adjusted mortality rate
AIAN: American Indian or Alaskan native
APC: Annual percent change
API: Asian or Pacific Islander
CDC WONDER: Centers for disease control and prevention wide-ranging ONline data for epidemiologic research
HF: Heart failure
ICD: International classification of diseases
NH: Non-Hispanic
NHB: Non-Hispanic black
NHW: Non-Hispanic white
